# Implication of quantifying nitrate utilization and CO_2_ assimilation of *Brassica napus* plantlets in vitro under variable ammonium/nitrate ratios

**DOI:** 10.1186/s12870-022-03782-8

**Published:** 2022-08-06

**Authors:** Kaiyan Zhang, Yanyou Wu, Yue Su, Haitao Li

**Affiliations:** 1 School of Karst Science, Guizhou Normal University/State Engineering Technology Institute for Karst Desertification Control, Guiyang, 550001 China; 2 State Key Laboratory of Environmental Geochemistry, Institute of Geochemistry, Chinese Academy of Sciences, No. 99 Lincheng West Road, Guanshanhu District, Guiyang, Guizhou Province 550081 People’s Republic of China; 3 Department of Agricultural Engineering, Guizhou Vocational College of Agriculture, Qingzhen, 551400 China

**Keywords:** Ammonium, Bidirectional stable isotope tracer, Isotope mixing model, Nitrate, Nitrogen assimilation, Nitrogen use efficiency

## Abstract

**Background:**

Plantlets grown in vitro with a mixed nitrogen source utilize sucrose and CO_2_ as carbon sources for growth. However, it is very difficult to obtain the correct utilization proportions of nitrate, ammonium, sucrose and CO_2_ for plantlets. Consequently, the biological effect of ammonium/nitrate utilization, the biological effect of sucrose/CO_2_ utilization, and the ammonium/nitrate use efficiency for new C input derived from CO_2_ assimilation/sucrose utilization are still unclear for plantlets.

**Results:**

The bidirectional stable nitrogen isotope tracer technique quantified the proportions of assimilated nitrate and ammonium in *Brassica napus* plantlets grown at different ammonium/nitrate ratios. The utilization proportions of sucrose and CO_2_ could be quantified by a two end-member isotope mixing model for *Bn* plantlets grown at different ammonium/nitrate ratios. Under the condition that each treatment contained 20 mM ammonium, the proportion of assimilated nitrate did not show a linear increase with increasing nitrate concentration for *Bn* plantlets. Moreover, the proportion of assimilated CO_2_ did not show a linear relationship with the nitrate concentration for *Bn* plantlets. Increasing the nitrate concentration contributed to promoting the assimilation of ammonium and markedly enhanced the ammonium utilization coefficient for *Bn* plantlets. With increasing nitrate concentration, the amount of nitrogen in leaves derived from nitrate assimilation increased gradually, while the nitrate utilization coefficient underwent no distinct change for *Bn* plantlets.

**Conclusions:**

Quantifying the utilization proportions of nitrate and ammonium can reveal the energy efficiency for N assimilation in plantlets grown in mixed N sources. Quantifying the utilization proportion of CO_2_ contributes to evaluating the photosynthetic capacity of plantlets grown with variable ammonium/nitrate ratios. Quantifying the utilization proportions of nitrate, ammonium, sucrose and CO_2_ can reveal the difference in the ammonium/nitrate use efficiency for new C input derived from CO_2_ assimilation/sucrose utilization for plantlets grown at variable ammonium/nitrate ratios.

**Supplementary Information:**

The online version contains supplementary material available at 10.1186/s12870-022-03782-8.

## Background

Nitrate and ammonium are widely used in plant tissue culture. From the view of the energy cost of nitrogen (N) assimilation, ammonium might be regarded as the preferable N source because the nitrate reduction that reduces nitrate to ammonium consumes large amounts of reducing power [1]. However, excessive ammonium has detrimental effects on plant growth (ammonium toxicity) [1–3]. In general, ammonium toxicity can be alleviated by adding a small amount of nitrate [1, 2]. Hence, a combination of an appropriate nitrate concentration and a high concentration of ammonium will contribute to reducing the energy cost used for nitrogen assimilation.

Murashige and Skoog (MS) [4] medium, which has a high inorganic nitrogen concentration (60 mM), is widely used for most plant species. However, for some plant cultures, the amount of inorganic N in MS medium far exceeds the amount required for normal growth of plantlets in vitro [5, 6]. In addition, the ratio of ammonium to nitrate (1:2) in MS medium might not be optimal because Zhang et al. [6] found that the nitrogen in the leaves of plantlets was mainly derived from ammonium assimilation even if the concentration of nitrate was twice that of ammonium. Hence, excessive nitrate in MS medium is not optimal and causes a waste of inorganic N. Considering the fact that the concentration of nitrate was twice that of ammonium in MS medium (20 mM ammonium, 40 mM nitrate), optimizing the nitrate concentration will effectively improve the nitrogen use efficiency of plantlets. Moreover, when the ammonium concentration is fixed at 20 mM in culture medium, optimizing the nitrate concentration can provide a chance to understand the reason for the reported relief of nitrate-dependent ammonium toxicity.

During the multiplication stage, most plantlets utilize sucrose (usually 3% in MS medium, w/v) and CO_2_ as carbon (C) sources for mixotrophic growth, i.e., CO_2_ for autotrophic growth and sucrose for heterotrophic growth [7]. Hence, the new C input in plantlets is derived from CO_2_ assimilation and sucrose utilization. The proportion of assimilated CO_2_ can indicate the degree of photoautotrophy (i.e., the photosynthetic capacity) for plantlets [7]. Generally, the survival rate of plantlets during acclimation is positively correlated with their photosynthetic capacity [8]. Moreover, high photosynthetic capacity is usually accompanied by the production of more reducing power, which contributes to enhancing the assimilation of nitrate and ammonium. The growth status of plantlets is closely associated with their photosynthetic capacity. Hence, quantifying the proportion of assimilated CO_2_ will contribute to evaluating the photosynthetic capacity of plantlets.

The plant C/N ratio can be employed as a proxy measure of N-use efficiency (NUE) over time [9]. Generally, the C and N contents in leaves can be measured directly. Therefore, when the utilization proportions of nitrate, ammonium, sucrose and CO_2_ are known for plantlets, the C content derived from the CO_2_ assimilation/sucrose utilization can be obtained; the N content derived from the assimilation of nitrate/ammonium can also be obtained. As a result, the nitrate/ammonium use efficiency for the new C input derived from CO_2_ assimilation/sucrose utilization can be represented by the corresponding C/N ratio, which contributes to revealing the difference in the ammonium/nitrate use efficiency for new C input derived from CO_2_ assimilation/sucrose utilization for plantlets grown at variable ammonium/nitrate ratios. However, it is very difficult to quantify the utilization proportions of nitrate, ammonium, sucrose and CO_2_ by chemical methods.

The nitrogen isotope composition (δ^15^N) of plants is strongly connected to the δ^15^N of the culture substrate [10, 11]. Therefore, plant δ^15^N is widely used as an indicator of nitrogen sources [12–14]. Both nitrate reductase (NR) and glutamine synthetase (GS) discriminate against ^15^ N relative to ^14^ N [9, 15]. Hence, nitrogen isotope fractionation occurs during the assimilation of nitrate and ammonium. The nitrogen isotope discrimination of NR is in the range of 19–22‰ [15–17] or 26‰ [18], whereas the nitrogen isotope fractionation value of GS is 16.5 ± 1.5‰ [19]. The assimilation of inorganic nitrogen occurs in the roots and/or shoots depending on the plant species and available N form [20, 21]. As a result, the nitrogen isotope fractionation values of nitrate assimilation and ammonium assimilation are also difficult to obtain simultaneously. Hence, quantifying the proportion of assimilated nitrate and ammonium is not possible with the δ^15^N of plants when a single isotope tracer is used at near-natural abundance levels. However, the assimilation of nitrate and ammonium only occurred in leaves in this study because the concentrations of cytokinin and auxin in this experiment precluded root formation by the plantlets. As a result, the foliar δ^15^N values of the root-free plantlets were only derived from the mix of the δ^15^N values of assimilated nitrate and ammonium in leaves without interference from the assimilation of nitrate and ammonium in the roots. Moreover, the cloned plantlets had no individual differences and were maintained in the same culture conditions in this study. Hence, based on the bidirectional stable N isotope tracer technique [6], the proportion of nitrate and ammonium utilization can be quantified in root-free plantlets when two labeled stable nitrogen isotope treatments (the *H* and *L* treatments) are used. The only difference between the *H* and *L* treatments is in the δ^15^N value of the nitrate, a δ^15^N of 22.67‰ in *H* and of 8.08‰ in *L*.

The stable carbon isotope technique is commonly used to identify various carbon sources utilized by plants [7, 22, 23]. Generally, a two end-member isotope mixing model can be used to quantify the utilization proportion of two different carbon sources [23, 24]. In this study, the growth of plantlets depended on the CO_2_ assimilation and sucrose utilization. Therefore, the foliar carbon isotopic composition (δ^13^C) of plantlets is derived from the mix of the δ^13^C values of assimilated CO_2_ and utilized sucrose_._ Accordingly, based on a two end-member isotope mixing model, the foliar δ^13^C values of plantlets can be used to quantify the utilization proportion of sucrose/CO_2_ if the isotope fractionation values of sucrose and CO_2_ are obtained.

In the present study, plantlets of *Brassica napus* (*Bn*), which is characterized by a very high demand for N inputs in agricultural systems [25], were subjected to different inorganic N regimes where the concentration of ammonium was set as 20 mM in each treatment. The following were our main aims: (1) to reveal the difference in nitrate utilization in *Bn* plantlets grown in variable ammonium/nitrate ratios and (2) to quantify the ammonium/nitrate use efficiency for new C input derived from CO_2_ assimilation/sucrose utilization for plantlets grown at variable ammonium/nitrate ratios.

## Methods

### Plant materials and experimental treatments

*Bn* plantlets in vitro were employed as explants in this experiment. Single shoots of *Bn* plantlets were grown in culture media with four inorganic nitrogen regimes. The average fresh weight (FW) per shoot was 0.09 g for the *Bn* plantlets. Based on the ammonium concentration (20 mM) in the MS culture medium, the ammonium concentration was set as 20 mM in each treatment, and the nitrate concentrations in the four treatments were set at 5 mM, 10 mM, 20 mM and 40 mM. Accordingly, the ammonium:nitrate ratio was different in each treatment. Each inorganic nitrogen regime included two labeled stable nitrogen isotope treatments. The labeled treatments were separated into groups with high (*H*) and low (*L*) natural ^15^ N abundance in NaNO_3_, with a δ^15^N of 22.67‰ in *H* and of 8.08‰ in *L*. NH_4_Cl, with a δ^15^N of -2.64‰, was employed as the ammonium nitrogen in this experiment. Each Erlenmeyer flask (150 ml) contained 50 ml Murashige and Skoog (MS) [4] medium supplemented with 2.0 mg·L^−1^ 6-benzylaminopurine, 0.2 mg·L^−1^ α-naphthylacetic acid, 3% (w/v) sucrose, and 7.5 g·L^−1^ agar. The Erlenmeyer flask was loosely closed with a piece of vented sealing film (vented membrane diameter available in 3 cm, pore size 0.2–0.3 μm), thus allowing gas exchange with the surrounding atmosphere. The concentrations of cytokinin and auxin in this experiment precluded root formation for *Bn* plantlets in vitro during the whole culturing stage. All culture media were adjusted to pH 5.8 and then autoclaved at 121 °C for 20 min. The *Bn* plantlets were maintained in a growth chamber with a 12-h photoperiod (50 μmol m^−2^ s^−1^ PPFD) at 25 ± 2 °C.

### Determination of growth parameters

After 5 weeks of culturing, the *Bn* plantlets were removed from the Erlenmeyer flasks in the afternoon. The biomass of each *Bn* plantlet (FW) was measured. Additionally, the leaf biomass of each *Bn* plantlet was also measured. Next, the shoots of each *Bn* plantlet were counted. The leaves of the *Bn* plantlets were dried at 60 °C. The increase in biomass of each plantlet (Increased biomass) was calculated as the difference between the initial FW of the shoot and the plantlet biomass after culture for 5 weeks. Moreover, the leaf dry weight (DW) of each *Bn* plantlet was also measured [see Additional file 1]. Finally, the dried leaves were ground to a fine powder.

### Chlorophyll concentration determination

A total of 0.1 g of fresh leaf that had been triturated in a mortar with a small amount of liquid nitrogen was macerated with 10 ml 95% ethanol for 24 h at 4 °C. The chlorophyll concentration in the extract was spectrophotometrically determined at 665 and 649 nm. The concentrations, including chlorophyll a and chlorophyll b concentrations, were determined on a fresh weight basis (mg·g^–1^) and calculated according to Alsaadawi et al. [26].

### Analysis of elements and determination of δ^15^N and δ^13^C in plantlets

The total nitrogen and carbon contents of the dried leaves were determined using an elemental analyzer (vario MACRO cube, Germany). Both δ^15^N and δ^13^C were measured by a gas isotope ratio mass spectrometer (MAT-253, Germany). Isotope ratios were calculated as follows:

1 $$\updelta {\left[{}^{13}\mathrm{C},{}^{15}\mathrm{N}\right]}_{\mathrm{samples}}=\left({R}_{\mathrm{sample}}/{R}_{\mathrm{standard}}-1\right)\times 1000$$

where *R*_sample_ refers to the ^13^C/^12^C or ^15^N/^14^ N of the plant material, and *R*_standard_ refers to the isotope ratio of a known standard (PDB or N_2_ in air). International isotope secondary standards of known ^13^C/^12^C ratios (IAEA CH_3_ and IAEA CH_6_) were used for calibration to a precision of 0.1‰. For nitrogen, isotope secondary standards of known ^15^N/^14^ N ratios (IAEA N_1_, IAEA N_2_, and IAEA NO_3_) were used to calibrate the instrument to reach a precision of 0.2‰ [27].

### Quantification of the contributions of nitrate and ammonium to total inorganic nitrogen assimilation

The proportions of nitrate and ammonium assimilated by *Bn* plantlets were determined by the bidirectional stable nitrogen isotope tracer technique [6]. Thus, the proportion of assimilated nitrate (*f*_A_) contributing to total inorganic nitrogen assimilation could be calculated by the following equation:

2 $${f}_{\mathrm{A}}=\left({\updelta }_{\mathrm{T}H}-{\updelta }_{\mathrm{T}L}\right)/\left({\updelta }_{\mathrm{A}H}-{\updelta }_{\mathrm{A}L}\right)$$

where δ_T*H*_ is the foliar δ^15^N value of the plantlets cultured with mixed-nitrogen sources, whose δ^15^N of nitrate in culture media was 22.67‰. δ_T*L*_ is the foliar δ^15^N value of the plantlets cultured with mixed-nitrogen sources, whose δ^15^N of nitrate in culture media was 8.08‰. Accordingly, δ_A*H*_ and δ_A*L*_ are the δ^15^N values derived from nitrate assimilation. The proportion of assimilated ammonium (*f*_B_) contributing to total inorganic nitrogen assimilation was calculated using the following equation:

3 $${f}_{\mathrm{B}}=1-{f}_{\mathrm{A}}$$

The standard error (SE) of *f*_A_ and *f*_B_ was achieved by the error propagation formula.

In this study, δ_T*H*_ and δ_T*L*_ could be obtained directly. However, when the plantlets were cultured in the medium with mixed-nitrogen sources, it would have been difficult to directly obtain δ_A*H*_ and δ_A*L*_, which are involved in nitrogen isotope discrimination in nitrate assimilation and the exchange of unassimilated nitrate between the shoot and the substrate during the whole culture period. Hence, δ_A*L*_ and δ_A*H*_ changed over time in this experiment. However, we were able to obtain δ_A*L*_ and δ_A*H*_ when the plantlets were grown in culture medium in which nitrate was the sole nitrogen source.

The δ_A*L*_ and δ_A*H*_ in nitrate-grown plantlets could be affected by unassimilated nitrate. However, a previous study found that the storage pool of nitrate in leaves of tomato and tobacco plants was replenished in the dark and became depleted in the light, and the foliar nitrate concentration of tomato and tobacco plants reached a low level in the afternoon [28, 29]. Hence, when the plantlets had been cultured for 5 weeks and harvested in the afternoon, the amount of unassimilated nitrate in the leaves of plantlets would be very small in comparison with the amount of assimilated nitrate. Moreover, the foliar δ^15^N value of *Bn* plantlets did not vary significantly among nitrate concentrations ranging from 10 to 40 mM [6, 30], which suggested that the effect of unassimilated nitrate in leaves on the foliar δ^15^N value could be neglected. As a result, the δ_A*L*_ and δ_A*H*_ of *Bn* plantlets grown in mixed-nitrogen sources could be replaced by the δ_A*L*_ and δ_A*H*_ in nitrate-grown *Bn* plantlets in this study.

Sodium nitrate with a δ^15^N of 22.67‰/8.08‰ was used as the sole nitrogen source in their study [6, 30]. Hence, the average foliar δ^15^N value in nitrate-grown *Bn* plantlets at the three nitrate supply levels (10, 20, and 40 mM) was approximately equal to the δ^15^*N* value (δ_A*L*_ or δ_A*H*_) of *Bn* plantlets cultured in the medium with mixed-nitrogen sources in this study. As a result, we were able to obtain δ_A*L*_ and δ_A*H*_. δ_A*L*_ was 3.17 ± 0.12‰ (*n* = 9, SE) for the *L Bn* plantlets [30], and δ_A*H*_ was 15.19 ± 0.29‰ (*n* = 9, SE) for the *H Bn* plantlets [6]. After determining δ_T*H*_, δ_T*L*_, δ_A*H*_ and δ_A*L*_, we were able to calculate *f*_A_ and *f*_B_. However, because the efflux of nitrate to the external media occurred during the whole culture period, we must acknowledge that end members δ_A*L*_ and δ_A*H*_ may change slightly if the proportional efflux of nitrate back to the media changes. In addition, based on the fact that the foliar δ^15^N value of *Bn* plantlets did not vary significantly among nitrate concentrations ranging from 10 to 40 mM [6, 30], the presence of ammonium was assumed to have no effect on net discrimination against nitrate in this study. Hence, the proportion of assimilated nitrate obtained by Eq. (2) might not be precise enough.

### Quantifying the contribution of nitrate/ammonium utilization to the amount of nitrogen in leaves

The nitrogen accumulation amount (NAA) of the leaves was the absolute nitrogen content in the dried leaves and was calculated using the following equation:

4 $$\mathrm{NAA}=\left(\mathrm{DW}\times \mathrm{Ncontent}\right)/\mathrm{M}$$

where M is the molar mass of nitrogen, and the N content of the dried leaves was determined by an elemental analyzer.

The nitrogen in leaves was derived from the assimilation of nitrate and ammonium. Therefore, the amount of nitrogen in leaves derived from assimilated nitrate/ammonium could be calculated by the following equations:

5 $${\mathrm{NAA}}_{\mathrm{nitrate}}=\mathrm{NAA}\times {f}_{\mathrm{A}}$$

6 $${\mathrm{NAA}}_{\mathrm{ammonium}}=\mathrm{NAA}\times {f}_{\mathrm{B}}$$

where NAA_nitrate_ is the amount of nitrogen in leaves derived from nitrate assimilation, and NAA_ammonium_ is the amount of nitrogen in leaves derived from ammonium assimilation. The standard error (SE) of NAA_nitrate_ and NAA_ammonium_ was calculated by the error propagation formula.

### Nitrogen utilization coefficient (NUC) of ammonium and nitrate

The nitrogen utilization coefficient (NUC) is the ratio of the total nitrogen content in the dried leaves relative to the nitrogen content in the medium. Therefore, the nitrogen utilization coefficient of ammonium (NUC_ammonium_) and nitrate (NUC_nitrate_) could be calculated by the following equation:

7 $${\mathrm{NUC}}_{\mathrm{ammonium}}\left(\mathrm{\%}\right)=\left({\mathrm{NAA}}_{\mathrm{ammonium}}/{\mathrm{n}}_{\mathrm{ammonium}}\right)\times 100$$

8 $${\mathrm{NUC}}_{\mathrm{nitrate}}\left(\mathrm{\%}\right)=\left({\mathrm{NAA}}_{\mathrm{nitrate}}/{\mathrm{n}}_{\mathrm{nitrate}}\right)\times 100$$

where n_ammonium_ and n_nitrate_ are the number of moles of ammonium and nitrate in the medium, respectively. The standard error (SE) of NUC_ammonium_ and NUC_nitrate_ was calculated by the error propagation formula.

### Quantifying the proportion of inorganic carbon utilization in *Bn* plantlets

In this study, the external C source apart from CO_2_ was the sucrose for *Bn* plantlets. Therefore, the foliar δ^13^C value of the *Bn* plantlet was derived from the mix of the δ^13^C values of assimilated inorganic and organic carbon. Based on a two end-member isotope mixing model [23, 24], an equation representing this utilization of two different carbon sources by *Bn* plantlets can be established as follows:

9 $${\updelta }_{\mathrm{T}}={f}_{\mathrm{P}}\times {\updelta }_{\mathrm{C}}+\left(1-{f}_{\mathrm{P}}\right)\times {\updelta }_{\mathrm{S}}$$

where δ_T_ is the foliar δ^13^C value of *Bn* plantlets grown in mixed-carbon sources and could be obtained directly. *f*_P_ is the proportion of assimilated CO_2_. 1-*f*_P_ is the proportion of utilized sucrose. δ_C_ is the δ^13^C value derived from CO_2_ assimilation. δ_S_ is the δ^13^C value derived from sucrose assimilation. Accordingly, Eq. (9) can be rewritten as Eq. (10):

10 $${f}_{\mathrm{P}}=\left({\updelta }_{\mathrm{T}}-{\updelta }_{\mathrm{S}}\right)/\left({\updelta }_{\mathrm{C}}-{\updelta }_{\mathrm{S}}\right)$$

The standard error (SE) of *f*_P_ was achieved by the error propagation formula.

When the plantlets were grown in mixed-carbon sources, it would have been difficult to directly obtain δ_S_ and δ_C_. To obtain δ_S_, the *Bn* plantlets were cultured in a CO_2_-free atmosphere where CO_2_ was absorbed by soda lime, and sucrose was the only carbon source for *Bn* plantlets. As a result, the isotope fractionation value of sucrose assimilation could be obtained indirectly. The isotope fractionation value of sucrose assimilation was 2.54 ± 0.13‰ (*n* = 3, SE) for *Bn* plantlets.

*Bn* plantlets cannot survive without a supply of sucrose. Hence, the isotopic fractionation value of CO_2_ assimilation cannot be obtained directly for *Bn* plantlets. To obtain δ_C_, the seeds of *Bn* were grown in MS culture medium (20 mM ammonium, 40 mM nitrate) without sucrose. The culture conditions for *Bn* seeds were exactly the same as those for the *L* and *H* treatments, i.e., they were grown at the same time in the same chamber in the same Erlenmeyer flasks, which were closed with the same vented sealing film. After 5 weeks of culturing, the leaves of *Bn* seedlings were harvested for the measurement of δ^13^C. The carbon in leaves was only derived from CO_2_ assimilation for *Bn* seedlings. Hence, the foliar δ^13^C value of the *Bn* seedling, derived from seed germination, could be used to approximate δ_C_ in this study. As a result, δ_C_ could be obtained indirectly and was -28.05 ± 0.13‰ (*n* = 3, SE) for *Bn* plantlets in this study. After δ_T_, δ_S_ and δ_C_ were known, we were able to calculate *f*_P_. However, the supply of inorganic N might affect the photosynthetic capacity of plants. Plants with low photosynthetic capacity might be more depleted in ^13^C than plants with high photosynthetic capacity. Hence, we must acknowledge that the δ_C_ in *Bn* plantlets grown at a concentration below 60 mM inorganic N might be less than -28.05‰ in this study. Hence, the *f*_P_ might be somewhat overestimated for *Bn* plantlets grown at a concentration below 60 mM inorganic N.

### The C/N ratios of leaves

After determining the carbon (C_T_) and nitrogen (N_T_) contents of leaves, the C_T_/N_T_ ratio of leaves could be obtained directly. In this study, we quantified the proportions of assimilated C and N in *Bn* plantlets. Accordingly, the carbon content derived from the CO_2_ assimilation (C_C_) and sucrose utilization (C_S_) could be obtained; the nitrogen content derived from the assimilation of nitrate (N_N_) and ammonium (N_A_) could also be obtained. As a result, the nitrate use efficiency for new C input derived from the CO_2_ assimilation (C_C_/N_N_ ratio), the nitrate use efficiency for new C input derived from sucrose utilization (C_S_/N_N_ ratio), the ammonium use efficiency for new C input derived from the CO_2_ assimilation (C_C_/N_A_ ratio), and the ammonium use efficiency for new C input derived from sucrose utilization (C_S_/N_A_ ratio) can be calculated by the following equations:

11 $${\mathrm{C}}_{\mathrm{C}}/{\mathrm{N}}_{\mathrm{N}} \mathrm{ratio }= \frac{{f}_{\mathrm{P} \times {\mathrm{C}}_{\mathrm{T}}}}{{f}_{\mathrm{A}} \times {\mathrm{N}}_{\mathrm{T}}} = \frac{{f}_{\mathrm{P}}}{{f}_{\mathrm{A}}} \times \frac{{\mathrm{C}}_{\mathrm{T}}}{{\mathrm{N}}_{\mathrm{T}}}$$

12 $${\mathrm{C}}_{\mathrm{S}}/{\mathrm{N}}_{\mathrm{N}} \mathrm{ratio} = \frac{\left(1-{f}_{\mathrm{P}}\right) \times {\mathrm{C}}_{\mathrm{T}}}{{f}_{\mathrm{A}} \times {\mathrm{N}}_{\mathrm{T}}} = \frac{1 - {f}_{\mathrm{P}}}{{f}_{\mathrm{A}}} \times \frac{{\mathrm{C}}_{\mathrm{T}}}{{\mathrm{N}}_{\mathrm{T}}}$$

13 $${\mathrm{C}}_{\mathrm{C}} /{\mathrm{N}}_{\mathrm{A}} \mathrm{ratio }= \frac{{f}_{\mathrm{P }\times {\mathrm{C}}_{\mathrm{T}}}}{\left(1 - {f}_{\mathrm{A}}\right) \times {\mathrm{N}}_{\mathrm{T}}} = \frac{{f}_{\mathrm{P}}}{1 - {f}_{\mathrm{A}}}\times \frac{{\mathrm{C}}_{\mathrm{T}}}{{\mathrm{N}}_{\mathrm{T}}}$$

14 $${\mathrm{C}}_{\mathrm{S}}/{\mathrm{N}}_{\mathrm{A}}\mathrm{ ratio}=\frac{\left(1-{f}_{\mathrm{P}}\right) \times {\mathrm{C}}_{\mathrm{T}}}{\left(1-{f}_{\mathrm{A}}\right) \times {\mathrm{N}}_{\mathrm{T}}}= \frac{1 - {f}_{\mathrm{P}}}{1 - {f}_{\mathrm{A}}} \times \frac{{\mathrm{C}}_{\mathrm{T}}}{{\mathrm{N}}_{\mathrm{T}}}$$

The standard error (SE) of the C_C_/N_N_ ratio, C_C_/N_A_ ratio, C_S_/N_N_ ratio, and C_S_/N_A_ ratio was achieved by the error propagation formula.

### Statistical analysis

The data were subjected to analysis of variance (ANOVA). The means of the different groups were compared via Tukey’s test (*p* < 0.05). The data are shown as the mean ± standard error (SE).

## Results

### Growth

The nitrate concentration had a significant effect on the growth of *Bn* plantlets. As shown in Table 1, when the ammonium concentration remained at 20 mM in each treatment, increasing the supply of nitrate could promote the growth of *Bn* plantlets. In addition, the leaf biomass of *Bn* plantlets increased significantly with increasing nitrate supply. With respect to the proliferation of shoots, the *Bn* plantlets showed no significant difference with increasing nitrate concentration, except at the lowest concentrations. Generally, *Bn* plantlets had good performance with respect to shoot proliferation under all treatments (Table 1).

**Table 1:** The growth parameters of *Brassica napus* plantlets cultured under nitrate treatment

Parameters	**NO**_**3**_**-N(mM) (+ 20 mM NH**_**4**_**-N)**			
	5	10	20	40
Increased biomass (g)	1.97 ± 0.26b	2.87 ± 0.25b	3.04 ± 0.28ab	4.20 ± 0.31a
Leaf biomass (g)	0.42 ± 0.05c	0.73 ± 0.06bc	0.89 ± 0.09ab	1.18 ± 0.07a
Number of shoots	4.7 ± 0.3b	7.3 ± 0.3a	7.3 ± 0.7a	7.7 ± 0.3a

Each nitrate treatment contained 20 mM ammonium. Each value represents the mean ± SE (*n* = 3). Values signed with the same letter in each line are not significantly different by Tukey’s test (*p* > 0.05)

### Chlorophyll concentrations

The chlorophyll concentration of the *Bn* plantlets was significantly affected by the nitrate supply. Under the condition that each treatment included 20 mM ammonium, the chlorophyll concentration of the *Bn* plantlets showed a positive response to increasing nitrate concentrations. Increasing the supply of nitrate could promote the biosynthesis of chlorophyll in *Bn* plantlets (Table 2).

**Table 2:** The chlorophyll concentration of *Brassica napus* plantlets cultured under nitrate treatment

Parameters	**NO**_**3**_**-N(mM) (+ 20 mM NH**_**4**_**-N)**			
	5	10	20	40
chl a (mg/g)	0.24 ± 0.02c	0.46 ± 0.03bc	0.64 ± 0.06ab	0.87 ± 0.09a
chl b (mg/g)	0.09 ± 0.01c	0.14 ± 0.01bc	0.20 ± 0.02b	0.29 ± 0.02a
chl a + b (mg/g)	0.32 ± 0.02c	0.60 ± 0.04bc	0.84 ± 0.08b	1.16 ± 0.10a

Each nitrate treatment contained 20 mM ammonium. Each value represents the mean ± SE (*n* = 3). Values signed with the same letter in each line are not significantly different by Tukey’s test (*p* > 0.05)

### Elemental analysis of the *Bn* plantlets

**Fig. 1:**
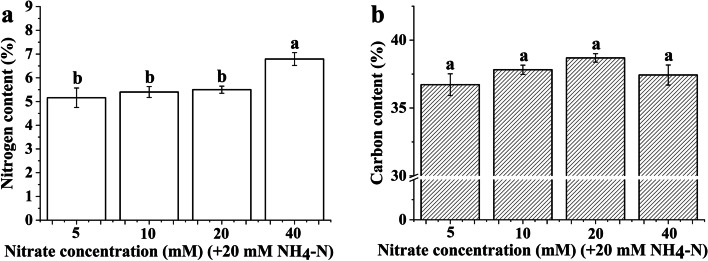
Nitrogen content (**a**) and carbon content (**b**) of the *Brassica napus* plantlets cultured under nitrate treatment. Each nitrate treatment contained 20 mM ammonium. The nitrogen and carbon content was expressed as a percent of foliar dry weight, respectively. The mean ± SE (*n* = 3) followed by different letters in the same legend differ significantly (Tukey’s test, *p* < 0.05)

The foliar nitrogen content of *Bn* plantlets was above 5% in all treatments. Supplying a certain concentration of nitrate could not effectively increase the foliar nitrogen content for *Bn* plantlets. As shown in Fig. 1, the foliar nitrogen content of *Bn* plantlets was not significantly different when the nitrate supply ranged from 5 to 20 mM. Moreover, the leaf carbon content of *Bn* plantlets did not show a significant difference with increasing nitrate (Fig. 1).

### Foliar carbon isotope ratio of the *Bn* plantlets

**Fig. 2:**
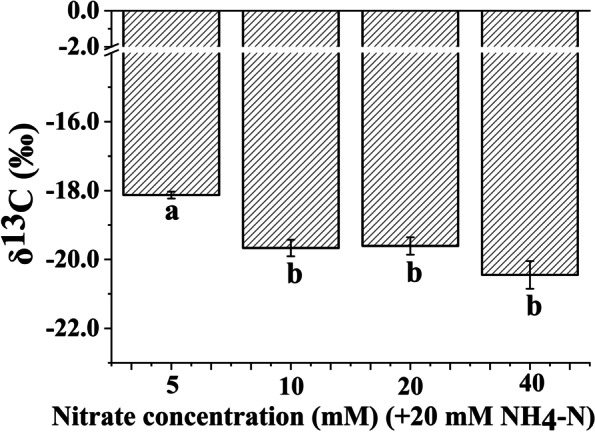
The δ^13^C values of *Brassica napus* plantlets in vitro cultured under nitrate treatment. Note: Each nitrate treatment contained 20 mM ammonium. The mean ± SE (*n* = 3) followed by different letters in the bar graph differ significantly (Tukey’s test, *p* < 0.05)

The δ^13^C values of *Bn* plantlets only showed significant differences at the lowest nitrate concentration. As shown in Fig. 2, increasing the nitrate supply did not significantly affect the δ^13^C values of *Bn* plantlets when the nitrate concentration was in the range of 10 to 40 mM.

### The proportion of CO_2_ and sucrose utilization by the *Bn* plantlets

**Fig. 3:**
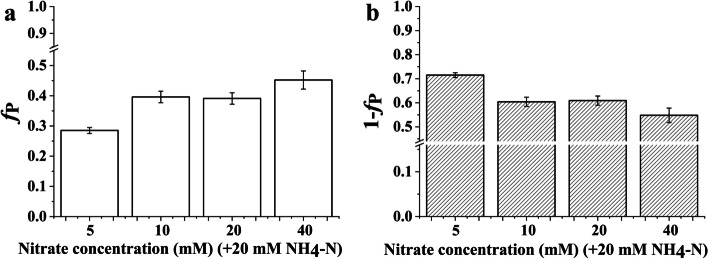
The proportion of CO_2_ (**a**) and sucrose (**b**) utilization by the *Brassica napus* plantlets cultured under nitrate treatment. Note: Each nitrate treatment contained 20 mM ammonium. The error bars were calculated by the error propagation formula

Increasing the supply of nitrate contributed to enhancing the proportion of CO_2_ utilization for *Bn* plantlets (Fig. 3). However, when the supply of nitrate reached 10 mM, there was no higher assimilation of CO_2_ for *Bn* plantlets. The proportion of CO_2_ utilization was lower than that of sucrose utilization at all nitrate concentrations, which suggested that the sucrose utilization was predominant for *Bn* plantlets. Nonetheless, increasing the nitrate concentration could reduce the predominance of the sucrose utilization.

### Foliar nitrogen isotope ratio of the *Bn* plantlets

**Fig. 4:**
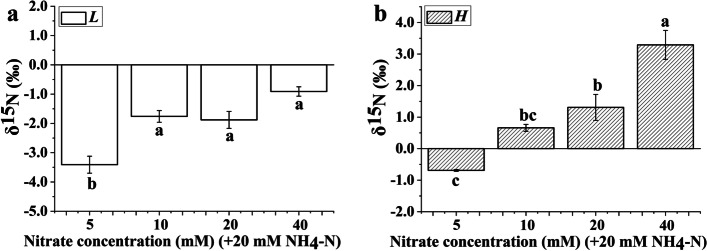
The foliar δ^15^*N* values of the *Brassica napus* plantlets cultured under nitrate treatment. Note: (**a**) The foliar δ^15^*N* values of the *Brassica napus* plantlets in the *L*-labeled treatment. (**b**) The foliar δ.^15^* N* values of the *Brassica napus* plantlets in the *H*-labeled treatment. Each nitrate treatment contained 20 mM ammonium. The mean ± SE (*n* = 3) followed by different letters in the same legend differ significantly (Tukey’s test, *p* < 0.05)

The δ^15^N values of *Bn* plantlets cultured in the *H* and *L* treatments were very different at different nitrate concentrations (Fig. 4). The minimum δ^15^N values in the *L* treatment were approximately -3.0‰, which suggested that δ^15^N values of the ammonium assimilation tended to impoverish in *Bn* plantlets. The δ^15^N value of *Bn* plantlets was significantly affected by nitrate concentration in both the *H* and *L* treatments. Increasing the supply of nitrate contributed to enriching ^15^N in *Bn* plantlets.

### The contribution of nitrate/ammonium to total inorganic nitrogen assimilation

**Fig. 5:**
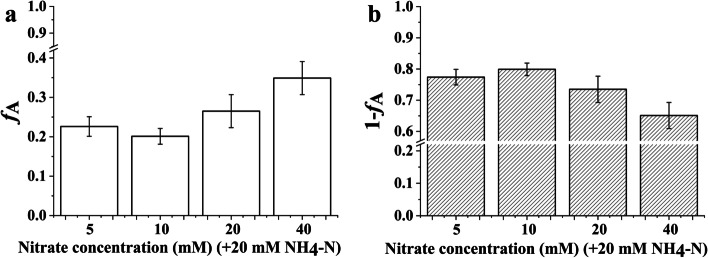
The contribution of nitrate (**a**) and ammonium utilization (**b**) to total inorganic nitrogen assimilation in the *Brassica napus* plantlets cultured under nitrate treatment. Note: Each nitrate treatment contained 20 mM ammonium. The error bars were calculated by the error propagation formula

The proportion of assimilated nitrate did not show a linear increase with increasing nitrate concentration for *Bn* plantlets (Fig. 5). The proportion of assimilated ammonium showed an obvious downward trend for *Bn* plantlets when the nitrate concentration increased from 10 to 40 mM. The contribution of nitrate utilization to total inorganic nitrogen assimilation was distinctly lower than that of ammonium in all treatments. Ammonium assimilation was predominant for *Bn* plantlets grown in mixed N source.

### The contribution of nitrate/ammonium utilization to the amount of nitrogen in leaves

**Fig. 6:**
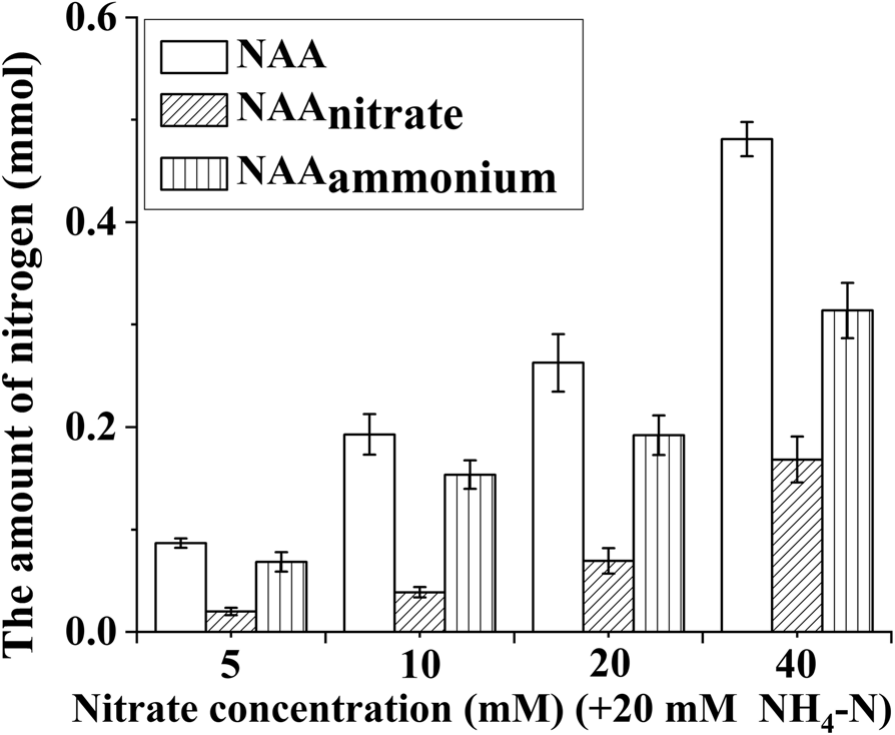
The amount of nitrogen in leaves (NAA), the amount of nitrogen in leaves derived from nitrate assimilation (NAA_nitrate_), and the amount of nitrogen in leaves derived from ammonium assimilation (NAA_ammonium_) in the *Brassica napus* plantlets cultured under nitrate treatment. Note: Each nitrate treatment contained 20 mM ammonium. The error bars of NAA_nitrate_ and NAA_ammonium_ were calculated by the error propagation formula

The amount of nitrogen in leaves (NAA) of *Bn* plantlets showed a positive response to increasing nitrate concentrations when each treatment contained 20 mM ammonium. As shown in Fig. 6, with increasing nitrate concentration, the amount of nitrogen in leaves derived from nitrate assimilation (NAA_nitrate_) gradually increased, and the amount of nitrogen in leaves derived from ammonium assimilation (NAA_ammonium_) also increased. Increasing the supply of nitrate could simultaneously promote the assimilation of nitrate and ammonium. NAA_ammonium_ increased more than double when the nitrate concentration reached 10 mM.

### The utilization coefficients of nitrate and ammonium of the *Bn* plantlets

**Fig. 7:**
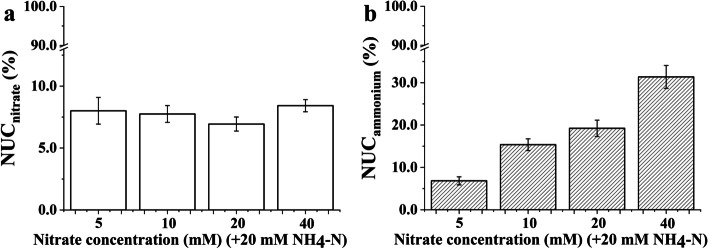
The utilization coefficients of nitrate (**a**) and ammonium (**b**) in the *Brassica napus* plantlets cultured under nitrate treatment. Note: Each nitrate treatment contained 20 mM ammonium. The error bars were calculated by the error propagation formula

The utilization coefficients of nitrate and ammonium of the *Bn* plantlets showed different responses to increasing nitrate concentrations when each treatment contained 20 mM ammonium. The nitrate utilization coefficients (NUC_nitrate_) of the *Bn* plantlets showed no distinct change with increasing nitrate concentration, while the ammonium utilization coefficients (NUC_ammonium_) of the *Bn* plantlets increased obviously with increasing nitrate concentration (Fig. 7). Increasing the supply of nitrate could markedly enhance the NUC_ammonium_ of the *Bn* plantlets, which contributed to reducing futile ammonium cycling.

### The C/N ratios of leaves

There were clear differences between the C_T_/N_T_ ratio, C_C_/N_N_ ratio, C_C_/N_A_ ratio, C_S_/N_N_ ratio, and C_S_/N_A_ ratio of leaves in *Bn* plantlets (Table 3). The C_T_/N_T_ ratio of leaves tended to decrease with 40 mM nitrate. The C_S_/N_N_ ratio and C_S_/N_A_ ratio of leaves showed a downward trend with increasing nitrate concentration.

**Table 3:** The C/N ratios of leaves in *Brassica napus* plantlets cultured under nitrate treatment

Parameters	**NO**_**3**_**-N(mM) (+ 20 mM NH**_**4**_**-N)**			
	5	10	20	40
C_T_/N_T_ ratio	7.22 ± 0.64	7.04 ± 0.34	7.05 ± 0.16	5.53 ± 0.27
C_C_/N_N_ ratio	8.96 ± 1.27	13.77 ± 1.61	10.38 ± 1.76	7.13 ± 1.02
C_C_/N_A_ ratio	2.62 ± 0.25	3.47 ± 0.24	3.75 ± 0.30	3.83 ± 0.39
C_S_/N_N_ ratio	22.49 ± 3.11	21.02 ± 2.34	16.13 ± 2.66	8.64 ± 1.19
C_S_/N_A_ ratio	6.58 ± 0.71	5.30 ± 0.39	5.83 ± 0.83	4.64 ± 1.13

Each nitrate treatment contained 20 mM ammonium. Each value represents the mean ± SE (*n* = 3). The standard error of the C_C_/N_N_ ratio, C_C_/N_**A**_ ratio, C_S_/N_N_ ratio, and C_S_/N_A_ ratio was calculated by the error propagation formula

With increasing nitrate concentration, the C_C_/N_N_ ratio of leaves first increased and then decreased, while the C_C_/N_A_ ratio of leaves slowly increased. As shown in Table 3, the C_S_/N_N_ ratio of leaves was higher than the C_S_/N_A_ ratio of leaves under each treatment; the C_C_/N_N_ ratio of leaves was also higher than the C_C_/N_A_ ratio of leaves under each treatment. Therefore, to some extent, the nitrate use efficiency for new C input was higher than that of ammonium.

## Discussion

Plant δ^15^N is a physiological indicator of N demand and fractionation that reflects changes in metabolic N fluxes and/or environmental effects [31, 32]. Since the use of nitrate and ammonium by plants are different, they exhibit different values of δ^15^N depending on the N source [33, 34]. In this study, the δ^15^N values of the *Bn* plantlets showed large differences between the *L*- and *H*-labeled treatments (Fig. 4). The foliar δ^15^N value of *Bn* plantlets is derived from the mix of the δ^15^N values of assimilated nitrate and ammonium in the leaves because no root formation occurs in this experiment. In addition, the δ^15^N values of *Bn* plantlets in each treatment are different from those of the substrate, which suggests that nitrogen isotope fractionation occurs during the assimilation of inorganic nitrogen in the *Bn* plantlets [30]. Generally, both the efflux of nitrate and ammonium to the external media and the assimilation of nitrate and ammonium can affect nitrogen isotope discrimination [35]. Therefore, if we are able to obtain the nitrogen isotope fractionation values of assimilated nitrate and ammonium, it will be possible to quantify the proportion of assimilated nitrate/ammonium with the δ^15^N values of the root-free plantlets in the *L*- or *H*-labeled treatments. However, it is very difficult to simultaneously obtain the nitrogen isotope fractionation values of nitrate assimilation and ammonium assimilation when the plantlets are grown in a mixed-nitrogen source.

According to the bidirectional stable nitrogen isotope tracer technique [6], when two labeled stable nitrogen isotope treatments are used, it is unnecessary to simultaneously obtain the nitrogen isotope fractionation values of nitrate assimilation and ammonium assimilation. As shown in Eq. (2), the proportion of assimilated nitrate depends only on δ_T*H*_, δ_T*L*_, δ_A*L*_ and δ_A*H*_. δ_T*H*_ and δ_T*L*_ are the foliar δ^15^N values of the *Bn* plantlets grown in the mixed-nitrogen source and can be obtained directly. δ_A*L*_ and δ_A*H*_ can be replaced by the foliar δ^15^N values of the plantlets grown in the corresponding culture medium in which nitrate is the sole nitrogen source. Hence, when δ_T*H*_, δ_T*L*_, δ_A*H*_ and δ_A*L*_ are determined, the proportion of assimilated nitrate/ammonium can be quantified for *Bn* plantlets. Meanwhile, the proportion of assimilated sucrose and CO_2_ can be quantified by Eq. (10) for *Bn* plantlets. As a result, the utilization proportions of nitrate, ammonium, CO_2_ and sucrose can be obtained simultaneously for *Bn* plantlets. The total nitrogen and carbon contents of leaves of *Bn* plantlets can be determined by an elemental analyzer in this study. Hence, the nitrate/ammonium use efficiency for new C input derived from CO_2_ assimilation/sucrose utilization can be represented by the corresponding C/N ratio for *Bn* plantlets [9].

Generally, an excessive nitrogen supply is usually accompanied by low nitrogen use efficiency [36, 37]. As shown in Table 3, the nitrate use efficiency for new C input derived from the CO_2_ assimilation (as indicated by the C_C_/N_N_ ratio) was the lowest for *Bn* plantlets when the nitrate concentration increased to 40 mM. Moreover, the nitrate use efficiency for new C input derived from sucrose utilization (as indicated by the C_S_/N_N_ ratio) was also the lowest for *Bn* plantlets when the nitrate concentration increased to 40 mM. These results indicate that excessive nitrate supply is not optimal for *Bn* plantlets. The excess of nitrate affects the assimilation of C, which results in a decrease in the nitrate use efficiency for new C input. Interestingly, the maximum nitrate use efficiency for new C input derived from the CO_2_ assimilation was not achieved at the lowest nitrate concentration for *Bn* plantlets. The C_C_/N_N_ ratio of *Bn* plantlets depended on the *f*_P_, *f*_A_, and C_T_/N_T_ ratios (Eq. 11) in this study. There was little difference in the C_T_/N_T_ ratio of *Bn* plantlets when the nitrate concentration was in the range of 5 to 20 mM. Hence, the C_C_/N_N_ ratio of *Bn* plantlets was mainly dependent on *f*_P_ and *f*_A_ when the nitrate concentration was in the range of 5 to 20 mM. As shown in Fig. 3, the *f*_P_ of *Bn* plantlets showed an obvious increase when the nitrate concentration increased from 5 to 10 mM. At the same time, the *f*_A_ of *Bn* plantlets was the lowest when the nitrate concentration was 10 mM (Fig. 5). As a result, the *Bn* plantlets obtained the maximum C_C_/N_N_ ratio when the nitrate concentration was 10 mM. Hence, an appropriate concentration of nitrate (10 mM) contributes to enhancing the C sink for *Bn* plantlets.

Under the condition that each treatment contained 20 mM ammonium, increasing the nitrate concentration contributed to elevating the proportion of assimilated nitrate for *Bn* plantlets when its concentration was in the range of 10 to 40 mM (Fig. 5). However, the maximum proportion of assimilated nitrate is only approximately 0.35 even if the concentration of nitrate is twice that of ammonium. These results indicate that the foliar nitrogen content of *Bn* plantlets is mainly derived from the assimilation of ammonium. The assimilation of ammonium is predominant among all treatments for *Bn* plantlets, which may be attributed to the lower energy cost for the assimilation of ammonium in comparison to the assimilation of nitrate [38, 39]. Generally, the assimilation of one nitrate molecule consumes 20 ATP, while the assimilation of one ammonium molecule only costs 5 ATP [38]. Consequently, the energy cost for the assimilation of 1 mol nitrogen will be in the range of 5 to 20 mol ATP for plantlets grown in a mixed N source. The proportion of assimilated nitrate reached the minimum value (approximately 0.2) for *Bn* plantlets when the nitrate concentration was 10 mM. As a result, we can conclude that the minimum energy cost of assimilating 1 mol nitrogen is approximately 8 mol ATP for *Bn* plantlets grown in the mixed N sources containing 20 mM ammonium. Hence, quantifying the proportion of assimilated nitrate and ammonium contributes to revealing the energy efficiency for N assimilation in plantlets grown in mixed N sources.

Plants usually suffer from ammonium toxicity when ammonium is supplied at high concentrations [2, 40]. However, ammonium toxicity can be alleviated by the addition of nitrate [1–3],or exogenous C sources [41, 42]. As shown in Table 1, under the condition that each treatment contained 20 mM ammonium, the increased biomass of *Bn* plantlets showed no significant change when the nitrate concentration was in the range of 5 to 20 mM. The *Bn* plantlets did not show apparent growth suppression when the nitrate concentration was only 5 mM, which might be related to the relatively high proportion of assimilated nitrate (Fig. 5). A recent study indicated that acidic stress caused by excessive ammonium assimilation is the primary cause of ammonium toxicity [43]. As shown in Fig. 1, the foliar nitrogen content of *Bn* plantlets was relatively high (above 5%) among all treatments. Hence, a relatively high proportion of assimilated nitrate contributes to alleviating acidic stress because the assimilation of nitrate is accompanied by the consumption of protons [43]. Moreover, an adequate supply of sucrose also led to the alleviation of ammonium toxicity in *Bn* plantlets [41, 42]. To some extent, increasing the nitrate concentration improves the growth of *Bn* plantlets. Therefore, C metabolism may be affected by the nitrate concentration in *Bn* plantlets.

The growth of *Bn* plantlets depended on the CO_2_ assimilation and sucrose utilization in this study. The proportion of assimilated CO_2_ can indicate the degree of photoautotrophy (i.e., the photosynthetic capacity) [7]. As shown in Fig. 3, the proportion of assimilated CO_2_ was obviously lower at the minimum nitrate concentration (5 mM) than at the other nitrate concentrations. The lowest proportion of CO_2_ assimilation indicates the weakest photosynthetic capacity. The poor photosynthetic capacity of *Bn* plantlets fed with the maximum ammonium/nitrate ratio (20 mM ammonium, 5 mM nitrate) may be attributed to the serious lack of chlorophyll because the low chlorophyll content in leaves usually limits the photosynthetic capacity of plants [44].

The chlorophyll content is usually positively correlated with the foliar nitrogen content because most leaf N is present in chloroplasts [45]. The foliar nitrogen content of *Bn* plantlets was relatively high (above 5%) and was almost the same when the nitrate concentration was in the range of 5 to 20 mM (Fig. 1). However, the chlorophyll content of *Bn* plantlets was obviously lower at 5 mM nitrate concentration than at the other nitrate concentrations (Table 2). The lowest chlorophyll content of *Bn* plantlets at 5 mM nitrate may be related to the acidic stress caused by excessive ammonium assimilation [43]. Acidic stress can cause a distinct decline in magnesium accumulation in plants [46]. Acidic stress can be significantly alleviated by an adequate supply of nitrate, whereas an insufficient supply of nitrate may not effectively alleviate acidic stress [47]. Hence, we speculate that the magnesium limit may inhibit the biosynthesis of chlorophyll when the nitrate concentration is only 5 mM.

With increasing nitrate concentration, the amount of nitrogen in leaves derived from nitrate assimilation (NAA_nitrate_) increases gradually (Fig. 6), which suggests that increasing the nitrate concentration enhances the assimilation of nitrate. Nitrate is mainly transported by NRTs, most of which are 2H^+^/1NO_3_^−^ symporters [48, 49]. Hence, increasing the nitrate concentration can alleviate the acidic stress caused by excessive ammonium assimilation. As a result, the biosynthesis of chlorophyll is improved due to the increased nitrate concentration (Table 2). A previous study found that an increased chlorophyll concentration usually leads to an increase in energy and reducing power [50]. However, we did not find that the photosynthetic capacity of *Bn* plantlets showed a linear relationship with the chlorophyll concentration. As shown in Fig. 3, the photosynthetic capacity of *Bn* plantlets was nearly the same when the nitrate concentration were 10 mM and 20 mM. The photosynthetic capacity of *Bn* plantlets did not increase when the nitrate concentration increased from 10 to 20 mM, which may be attributed to the elevated proportion of assimilated nitrate (Fig. 5). The increased proportion of assimilated nitrate consumes more energy and reducing power. As a whole, the increase in chlorophyll concentration may contribute to improving the photosynthetic capacity.

It is widely known that futile ammonium cycling occurs when ammonium is supplied at high concentrations, which results in a large energy loss [51–53]. The degree of futile ammonium cycling may be determined by the nitrate concentration [1]. Our results show that the assimilation of ammonium is obviously promoted by increasing nitrate concentrations (Fig. 6). The enhanced assimilation of ammonium leads to the reduction of futile ammonium cycling. Generally, excessive ammonium assimilation can promote acidic stress, which is considered to be the primary cause of ammonium toxicity [43]. However, increasing the supply of nitrate not only promotes the assimilation of ammonium, but also enhances the assimilation of nitrate (Fig. 6). The proportion of assimilated ammonium gradually decreased for *Bn* plantlets when the nitrate concentration increased from 10 to 40 mM. The nitrate reduction is accompanied by a consumption of H^+^ and leads to the production of a hydroxyl ion [38]. Hence, the alleviation of ammonium toxicity may be attributed to the reduction of futile ammonium cycling and the relief of acidic stress by the nitrate reduction process.

The effective coordination of C and N metabolism contributes to the optimal growth of plants [54]. Hence, the growth of *Bn* plantlets is improved when the nitrate concentration reaches 10 mM, which may be attributed to the minimum energy cost used for N assimilation per mole, the reduction of futile ammonium cycling, and the elevated photosynthetic capacity. In general, increasing the nitrate concentration contributes to improving the growth of *Bn* plantlets. Based on the effective management of inorganic N supply, it is necessary to know the utilization coefficients of nitrate and ammonium for plantlets grown in the culture media. Hence, quantifying the utilization coefficients of nitrate and ammonium contributes to optimizing the inorganic N supply for the plantlets. As shown in Fig. 7, the utilization coefficient of nitrate did not increase with increasing nitrate concentration. Meanwhile, the utilization coefficient of ammonium did not show an obvious increase when the nitrate concentration increased from 10 to 20 mM (Fig. 7). Furthermore, the photosynthetic capacity of *Bn* plantlets also did not increase when the nitrate concentration increased from 10 to 20 mM. Hence, given a basal concentration of 20 mM ammonium, the supply of 10 mM nitrate was the optimal combined concentration to improve N use efficiency for *Bn* growth.

## Conclusions

Based on the bidirectional stable nitrogen isotope tracer technique, the proportion of assimilated nitrate and ammonium can be quantified for *Bn* plantlets grown at variable ammonium/nitrate ratios. The minimum energy cost of assimilating 1 mol N is approximately 8 mol ATP for *Bn* plantlets grown in the mixed N sources containing 20 mM ammonium. The utilization proportion of sucrose and CO_2_ can be quantified by a two end-member isotope mixing model for *Bn* plantlets grown at variable ammonium/nitrate ratios. Quantifying the utilization proportions nitrate, ammonium, sucrose and CO_2_ contributes to revealing the difference in the ammonium/nitrate use efficiency for new C input derived from CO_2_ assimilation/sucrose utilization in plantlets grown at variable ammonium/nitrate ratios and provides a new insight that the nitrate-dependent alleviation of ammonium toxicity might be attributed to the stimulation of ammonium assimilation that would mitigate the futile ammonium cycling. We also postulate an enhancement of photosynthesis by nitrate and relief of acidic stress by the nitrate reduction process.

## Supplementary Information

**Additional file 1: Table S1**. The leaf dry weight of *Brassica napus* plantlets cultured under nitrate treatment.

## Data Availability

All data generated or analyzed during this study are included in this published article and its Additional file 1: Table S1.
