# Supplementary material for: Implication of quantifying nitrate utilization and CO2 assimilation of Brassica napus plantlets in vitro under variable ammonium/nitrate ratios
Source: BMC Plant Biol. 2022 Aug 6;22:392. doi: 10.1186/s12870-022-03782-8 (PMC9356413; doi:10.1186/s12870-022-03782-8)
Supplement: Supplementary file 1 — Additional file 1: Table S1. The leaf dry weight of Brassica napus plantlets cultured under nitrate treatment. [file 12870_2022_3782_MOESM1_ESM.docx]

Table S1 The leaf dry weight of *Brassica napus* plantlets cultured under nitrate treatment

| Parameters | NO_3_-N(mM) (+20 mM NH_4_-N) | | | |
| --- | --- | --- | --- | --- |
|  | 5 | 10 | 20 | 40 |
| Leaf dry weight (mg) | 24.0±2.6c | 49.9±3.8b | 66.6±5.2b | 99.4±4.2a |

Note: Each nitrate treatment contained 20 mM ammonium. Each value represents the mean ± SE (n=3). Values signed with the same letter in each line are not significantly different by Tukey’s test (p>0.05).
